# Totally Endoscopic Sublay Anterior Repair (TESAR) for diastasis recti, primary and incisional ventral hernias: Long-term clinical outcomes and quality of life

**DOI:** 10.1007/s10029-026-03808-2

**Published:** 2026-07-31

**Authors:** Federico Fiori, Francesco Ferrara, Carlo Corbellini, Lorenzo Gallitiello, Domenico Lo Conte, Marco Stella

**Affiliations:** 1 Department of Surgery, Division of General and Emergency Surgery, Unit of Abdominal Wall Surgery, “San Carlo Borromeo” Hospital, ASST Santi Paolo e Carlo, Milan, Italy; 2 Department of Precision Medicine in Medical, Surgical and Critical Care (Me.Pre.C.C.), University of Palermo, Palermo, Italy; 3 Department of Surgery, Unit of General and Oncological Surgery, “Paolo Giaccone” University Hospital, Palermo, Italy; 4 Department of Surgery, Division of General and Emergency Surgery, “San Carlo Borromeo” Hospital, ASST Santi Paolo e Carlo, Milan, Italy

**Keywords:** Ventral hernia, Diastasis recti, Minimally invasive surgery, Retromuscular repair, Quality of life

## Abstract

**Purpose:**

Abdominal midline defects, like diastasis recti (DR), primary and incisional ventral hernias, significantly impair function and quality of life. Minimally invasive abdominal wall reconstruction is increasingly shifting toward extraperitoneal approaches. This study aimed to evaluate the safety, efficacy, and long-term outcomes of Totally Endoscopic Sublay Anterior Repair (TESAR), an anterior completely extraperitoneal technique, through a retrospective observational analysis.

**Methods:**

Consecutive adult patients undergoing TESAR for DR and/or primary and/or incisional midline ventral hernias between January 2018 and March 2025 were included. The primary endpoint was the 30-day postoperative complication rate. Secondary endpoints included length of stay, recurrence, and quality of life (QoL), assessed by EuraHS-QoL and Carolinas Comfort Scale (CCS) up to 12 months. Recurrence was assessed clinically at scheduled visits or via telemedicine and by imaging (ultrasound or CT). The impact of technical refinements, including subcutaneous quilting suture, was analyzed.

**Results:**

A total of 120 patients were analyzed, with a median follow-up of 31 months. Indications included DR with umbilical hernia in 83 patients (69.2%), incisional hernias in 39 patients (32.5%), and other primary hernias in 15 patients (12.5%). No intraoperative complications occurred. Median follow-up was 31 months (range 12–81, IQR 18–54.5). Postoperative morbidity was 6.7% (seroma 5%, hematoma 1.6%), with no surgical site infections. No recurrences were clinically detected during follow-up. Quilting suture reduced clinically relevant seromas to 0%. Median hospital stay was 3 days. EuraHS-QoL scores improved from a median of 57 preoperatively to 0 at 12 months, and CCS scores from 35.5 to 0.

**Conclusion:**

TESAR appears to be a safe and effective minimally invasive technique for selected patients, associated with low morbidity and significant improvement in QoL. However, given the retrospective, observational and non-comparative design, these findings should be interpreted cautiously. Further prospective comparative studies are warranted.

**Supplementary Information:**

The online version contains supplementary material available at 10.1007/s10029-026-03808-2.

## Introduction

Midline ventral hernias, either primary or incisional, and diastasis recti (DR) are common and often symptomatic conditions that impair quality of life through pain, functional limitations, and body-image concerns [1, 2]. Minimally invasive repair has historically been dominated by intraperitoneal onlay mesh (IPOM), with or without fascial closure (IPOM-plus) [3, 4]. While IPOM reduces wound morbidity compared with open surgery, intraperitoneal mesh placement exposes patients to mesh–viscera interactions and potential long-term mesh-related bowel events. Moreover, IPOM has been challenged by postoperative bulging and chronic pain in some series and other complications, such as occlusion and perforation [5–7]. In response, multiple extraperitoneal approaches have emerged. Posterior extraperitoneal retromuscular repairs, in particular the enhanced-view Totally Extraperitoneal (eTEP) and its derivatives, attempt to endoscopically recreate a Rives-Stoppa concept [8–11], whereas the Preperitoneal enhanced-view Totally Extraperitoneal (PeTEP), introduced in 2023 by the group of Dr. Valenzuela, uses direct preperitoneal endoscopic access by completing the preperitoneal dissection and placing the mesh posterior to the rectus sheath in the preperitoneal space [12]. In parallel, a large family of anterior endoscopic subcutaneous approaches (SCOLA/REPA/EPAR and related variants [13–19]) has proliferated for DR and small midline hernias; these approaches are generally reinforced by onlay mesh but are frequently complicated by seroma due to wide preaponeurotic dissection [13]. Totally Endoscopic Sublay Anterior Repair (TESAR) was introduced by our group in 2019 as a different approach: using safe anterior suprapubic endoscopic access while achieving true retromuscular (retrorectus) sublay mesh repair [20]. This approach aims to combine the low visceral risk and ergonomic advantages of anterior endoscopy (easy dissection of the working space, no intraperitoneal entry, no “en reverse” posterior suturing without a robotic platform, easy learning curve, easy management of the hernia sac, better skin retraction) with the biomechanical and infection resistance benefits of the retromuscular plane [20–23].

The present study reports the short- and long-term outcomes of TESAR performed by our group, focusing on postoperative complications and long-term results in terms of recurrence and quality of life, with a comparison with currently available minimally invasive abdominal wall reconstruction techniques.

## Materials and methods

This is an observational retrospective study including consecutive patients who underwent TESAR and were enrolled between January 2018 and March 2025. The primary indication for TESAR are midline ventral defects, that in this cohort was mainly represented by symptomatic DR associated with umbilical and other midline hernias, followed by isolated primary or incisional midline hernias. Patient-level variables were extracted from a prospectively maintained database. All patients underwent preoperative studies of the abdominal wall with physical and radiological examinations, ultrasound (US) and/or computed tomography (CT). The inclusion criteria were age *≥* 18 years, BMI < 35 kg/m2, the presence of at least one primary and/or incisional midline defect and/or DR > 50 mm, with an umbilical hernia, detected on US or CT scan, and for female patients more than 1 year from the last delivery and no intention of further pregnancy. Pregnant patients, patients within 1 year from the last pregnancy, cancer patients, and patients with general contraindications to laparoscopy were excluded. This study was conducted in accordance with the Declaration of Helsinki. Written informed consent was obtained from all individual participants included in the study.

The primary endpoint of this study was the 30-day post-operative complication rate, which included clinically relevant seroma (CRS), surgical site infection (SSI), and bleeding/hematoma. The secondary endpoints were the length of hospital stay (LOS), hernia and/or DR recurrence at the last available follow-up, and quality of life (QoL) at 1, 6 and 12 months after surgery.

CRS was defined as a postoperative abnormal accumulation of serous fluid requiring medical intervention or causing significant symptoms, such as pain, functional limitations, or wound leakage [24]. SSI was defined clinically and/or microbiologically when reported. Recurrence was defined as a clinically relevant bulge confirmed by imaging when available. The complications were classified according to the Clavien‒Dindo classification [25].

Follow-up was assessed at 1 week after discharge and at 1, 3, 6, and 12 months and, when possible, every 12 months after the operation via a clinical and, in some cases, radiological checkup. If the physical examination was not possible, the checkup was performed via telemedicine (mobile app with photo and/or video evaluation) and always associated with radiological examination (ultrasound or CT).

QoL was measured via two different tools: the EuraHS-QoL and the Carolinas Comfort Scale (CCS) [26, 27]. The EuraHS-QoL tool is a hernia-specific questionnaire with nine questions that can be scored by the patient on an 11-point scale from 0 to 10. The EuraHS-QoL questions are divided into three domains: “Pain” (range 0–30), “Restriction of activities” (range 0–40), and “Esthetic discomfort” (range 0–20). The total score ranges from 1 3 0 to 90, with lower scores being the most favorable outcome. In our study, the EuraHS-QoL score was measured preoperatively and at 7 days, 1 month, 6 months and 12 months. The EuraHS-QoL score was reported as the median value. The total score (nine questions), pain domain (three questions), restrictions domain (four questions), and cosmetic domain (two questions) were reported. The sum of the scores for the different questions in each domain and overall was calculated. The CCS is a well-studied and validated disease-specific questionnaire for patients who undergo mesh hernia repair; it uses a 23-item questionnaire designed to assess QoL and pain related to mesh abdominal hernia repair by measuring patient comfort across eight daily activities—such as walking, bending, and coughing—using a 6-point Likert scale, with total scores ranging from 0 to 115. In our study, the CCS was measured preoperatively and at 1 month, 6 months and 12 months. The median values of the total CCS score for each domain were reported and compared.

### Surgical technique

**Fig. 1:**
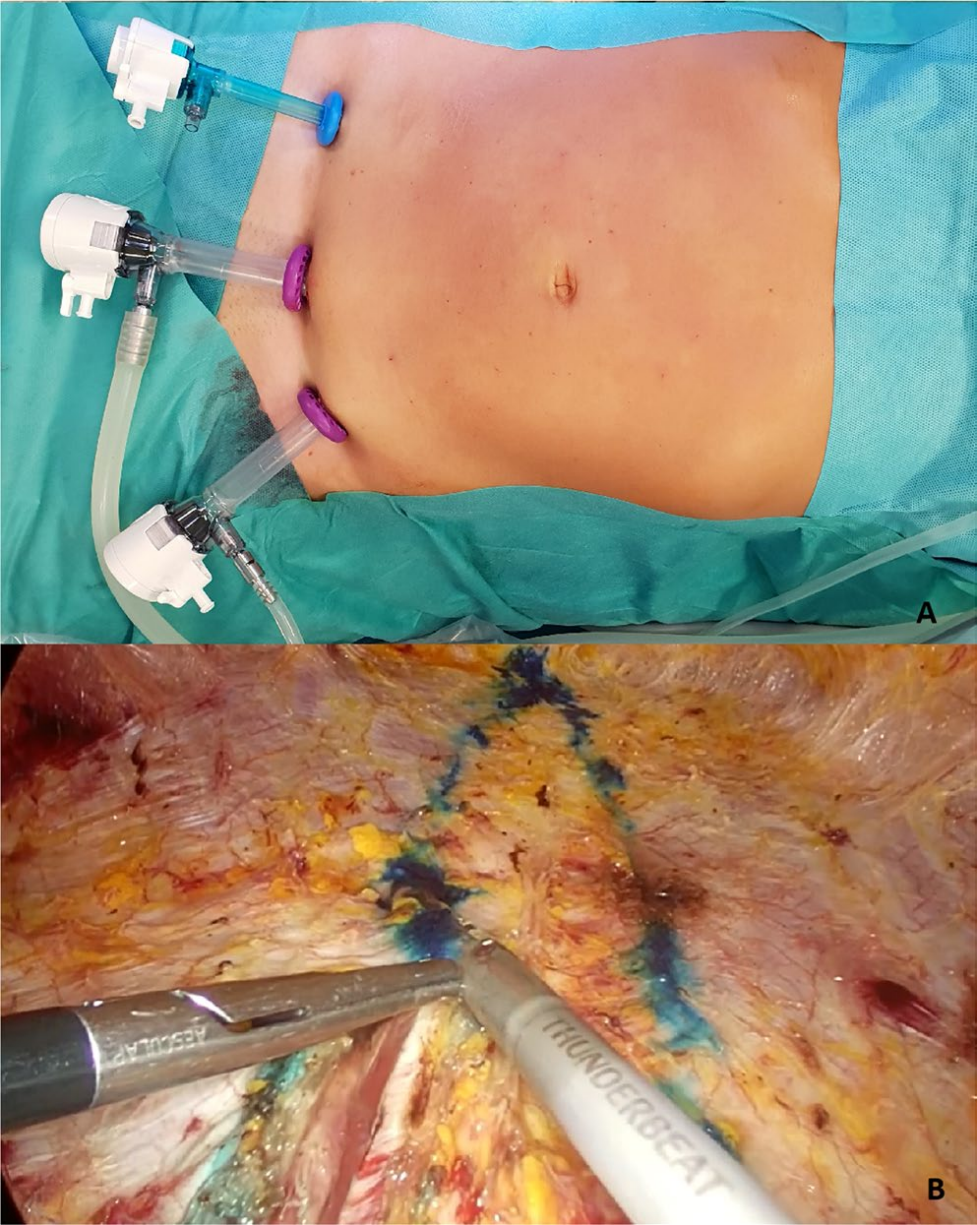
TESAR for diastasis recti: trocar positioning (**A**) and defect isolation (**B**)

**Fig. 2:**
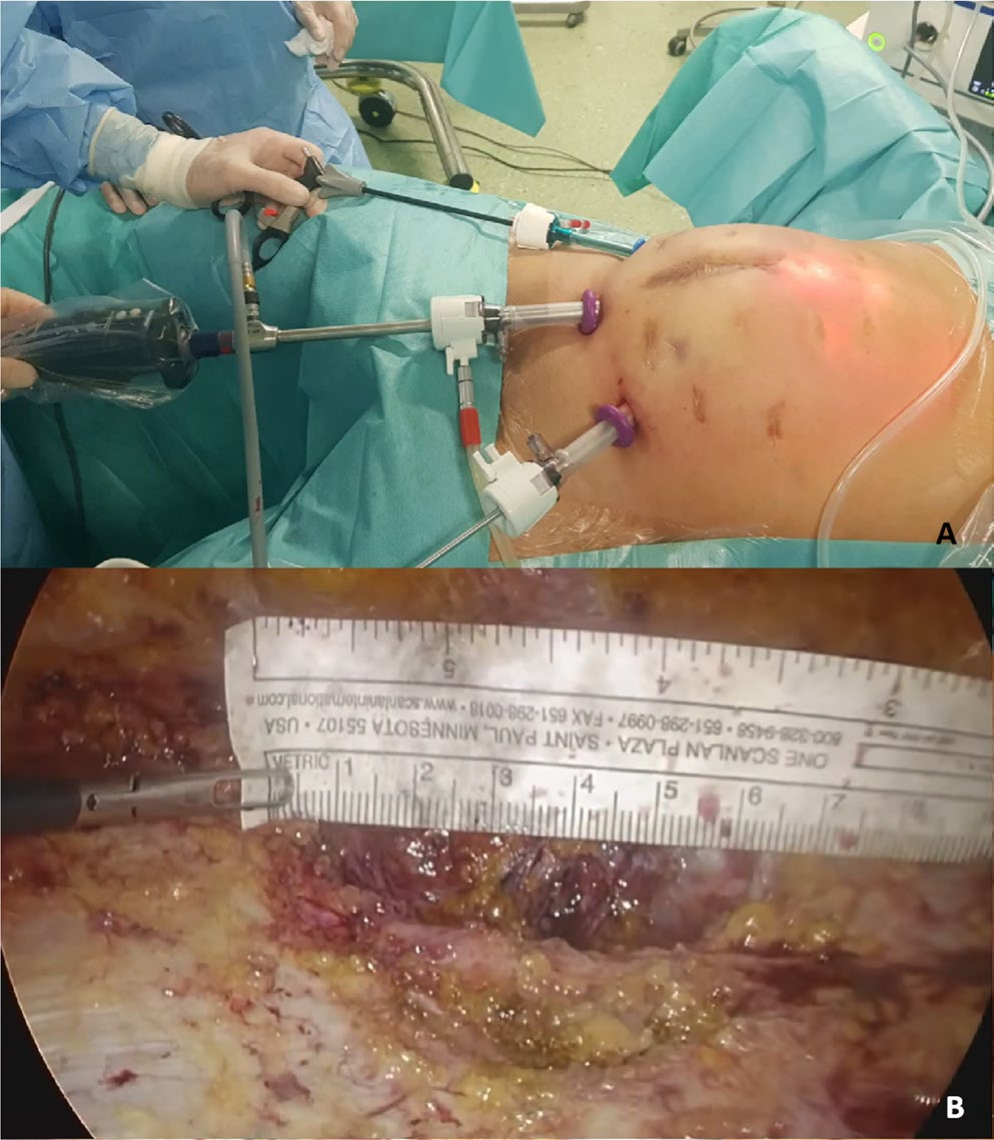
TESAR for incisional hernia: trocar positioning (**A**) and defect isolation (**B**)

**Fig. 3:**
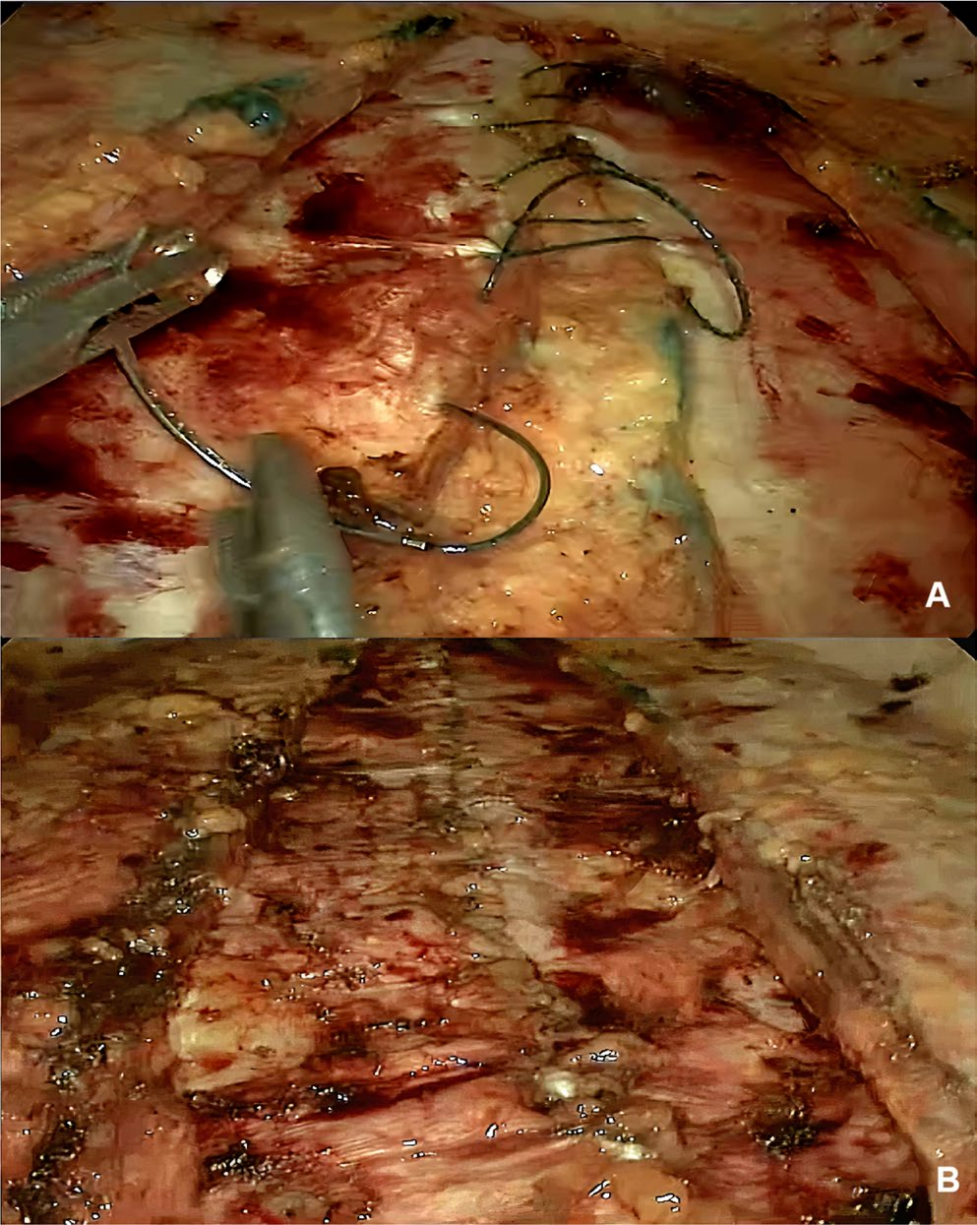
posterior rectus sheath plication (**A**-**B**)

**Fig. 4:**
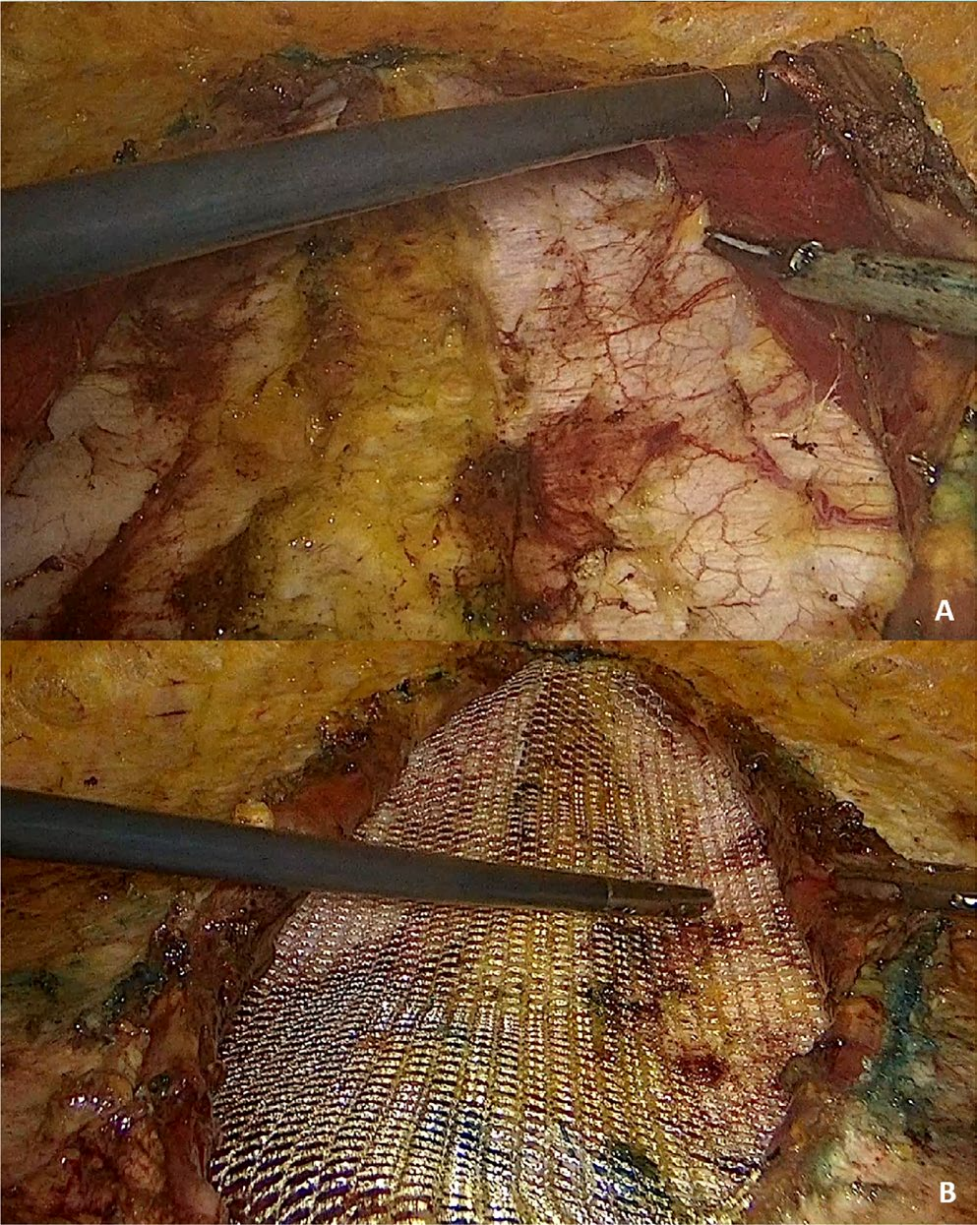
retromuscular dissection (**A**) and sublay mesh positioning (**B)**

**Fig. 5:**
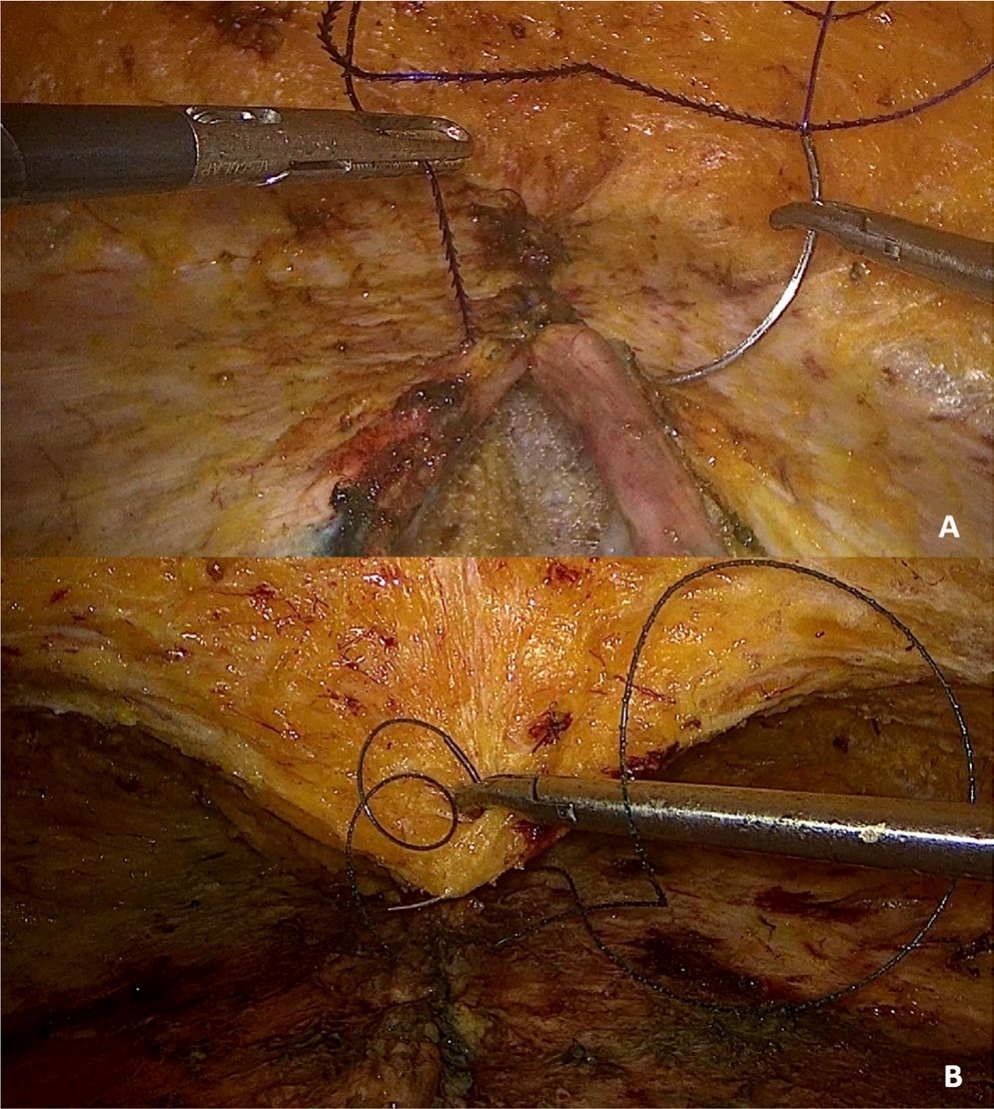
midline closure with sublay mesh (**A)** and subcutaneous quilting suture (**B**)

TESAR, introduced by our group, has already been described for the treatment of ventral and incisional hernias, as well as DR [20, 23]. This technique combines preaponeurotic endoscopic dissection with retromuscular repair. The operation can be conceptualized as two spaces: (a) a preaponeurotic/subcutaneous working space used to expose the linea alba and the defects and (b) a retrorectus (retromuscular) space used for definitive mesh placement. A 1 cm suprapubic access is realized, with a camera port placed in the midline and two working ports laterally **(**Fig. 1 A**)**. The preaponeurotic plane is developed cranially under direct endoscopic visualization toward the xiphoid, dissecting the umbilicus from the fascia and maintaining meticulous hemostasis to minimize bleeding into this space. The hernia sac is reduced and, when appropriate, partially excised. Complete exposure of the linea alba facilitates precise identification of the hernia margins and the diastasis segment **(**Figs. 1B and 2B**)**. This step is also critical for morphological correction, especially in thin patients with a visible bulge. The medial margins of both anterior rectus sheaths are incised bilaterally, exposing the rectus muscles. The posterior layer is preserved whenever possible. If we need to reload the transversus muscles to gain muscular function, sometimes the posterior sheath can be plicated without risk **(**Fig. 3**)**. Through this window, the retrorectus plane is entered and developed bilaterally to create a continuous retromuscular space. Definitive reinforcement is performed by placing a large mesh in the retrorectus plane **(**Fig. 4**)**. Mesh fixation can be minimal or selective depending on the mesh type and tissue quality, and this can be done with fibrin or cyanoacrylate glue or sometimes with a single loose cranial stitch to avoid any translational movement. The anterior rectus sheaths are then closed in the midline, recreating the linea alba, restoring the midline tension vectors and isolating the mesh from the preaponeurotic compartment **(**Fig. 5A**)**. If indicated, the external oblique muscles can be plicated to increase muscular tension and reduce the abdominal core circumference. A fascial plication can also be performed transversely at the umbilical level to reduce the cranio-caudal fascial relaxation and compensate for the possible presence of the periumbilical muscular protrusion. One or two closed-suction drains are used at the discretion of the surgeon: one in the retromuscular space, one in the subcutaneous space, or both in the subcutaneous space. Because of preaponeurotic dissection, dead space management is critical for preventing seroma, so a specific technique modification was introduced to mitigate this complication during the study period. A subcutaneous-to-fascia quilting suture along the midline (progressive tension suture) is then used to attach the subcutaneous tissue to the anterior fascia over the dissected area, thereby collapsing the potential space and distributing tension [28] **(**Fig. 5B**)**. If indicated, following large dissections due to the need of plication of the oblique muscles, this suture can also be performed along the lateral margin of the rectus muscles. The technical steps of the procedure are the same for all the defect types. An exploratory pre/post analysis was performed in this study. The post-refinement period was defined as surgeries performed on or after April 26, 2023, corresponding to the first day of introduction of quilting suture in our surgical practice.

### Statistical analysis

Nominal data are expressed as frequencies and were compared via Pearson’s chi-square test and Fisher’s exact test. Numerical data are expressed as medians, and they were compared via the Mann–Whitney U test and Wilcoxon rank test. Continuous variables were compared via Pearson’s R correlation coefficient test. Given the small size of some subgroups, multiple confounders that have not been adjusted for, the sample size calculation not being appropriate and hence a possible lack of power, we decided that statistical significance would be reached only if the p value was < 0.05. Jamovi software (Version 2.6) was used for statistical analysis.

## Results

**Fig. 6:**
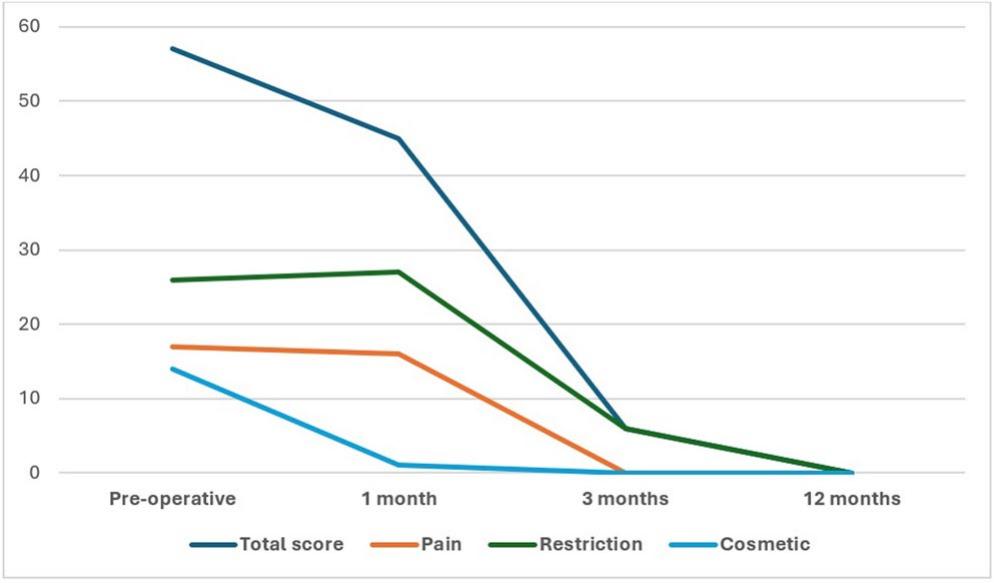
EuraHS-QoL scores variation

**Fig. 7:**
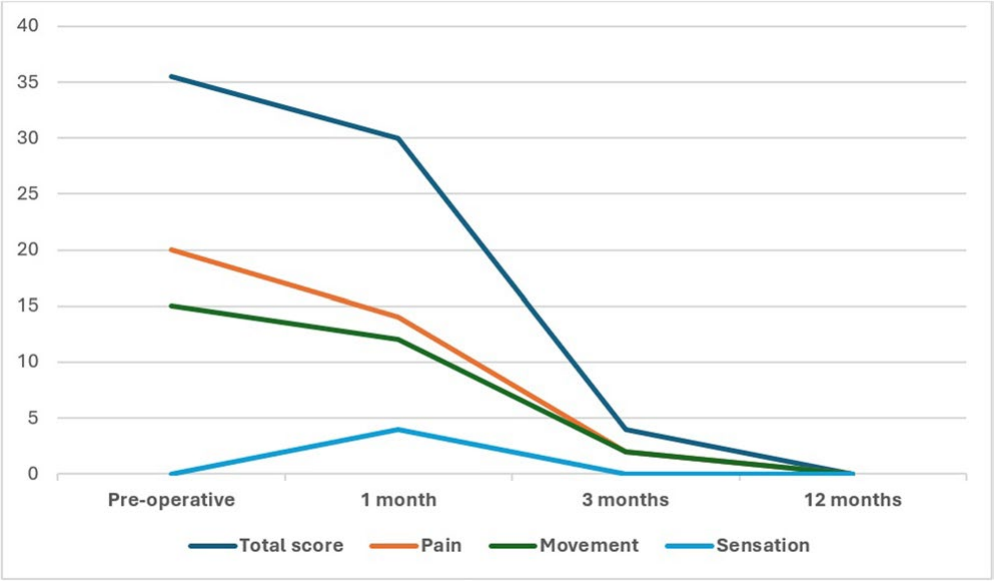
Carolinas Comfort Scale scores variation

Between January 2018 and March 2025, 120 patients underwent TESAR for surgical repair of midline ventral hernias, either primary or incisional, and/or DR. As shown in Table 1, the cohort consisted of 96 females (80%) and 24 males (20%), with a median age of 42 years (range 23–82) and a median BMI of 23.0 kg/m² (range 17.1–34.0). Previous abdominal operations were reported in 45.8% of patients. DR and umbilical hernias were present in 69.2% of the patients, incisional hernias were identified in 39 patients (32.5%), and other primary ventral hernias were identified in 15 patients (12.5%). The median operative time was 183 min (range 118–420) (Table 2). Transversus abdominis release (TAR) was performed in 8 patients (6.7%). A polypropylene mesh was implanted in 80.8% of the cases, whereas a poly-4-hydroxybutyrate mesh (P4HB) was used in 19.2% of the cases. Drain removal occurred at a median of 3 days for the first drain and 8 days for the second drain. Postoperative hospitalization lasted a median of 3 days (range 2–10). The median follow-up period was 31 months (range 12–81, IQR 18–54.5), with 18.3% of patients exceeding 60 months of follow-up (Table 3). A minimum of 12 months of follow-up was assessed in all patients. The overall postoperative morbidity rate was 6.7%, including Clavien–Dindo grade I (0.8%), grade II (0.8%), grade IIIa (4.2%), and grade IIIb (0.8%). Seroma was the most frequent complication, occurring in 6 patients (5%) who never needed surgical reoperation, whereas hematoma was observed in 2 patients (1.6%), one of whom needed surgical revision. Notably, no surgical site infections were recorded. As specified above, follow-up was conducted through a combination of in-person visits and telemedicine/radiological assessments. A total of 92 patients (76.7%) completed follow-up with direct clinical visit (42 patients underwent also radiological examination), whereas 28 patients (23.3%) were evaluated via telemedicine and radiological evaluation (US, CT). No recurrences were clinically detected or documented during follow-up. An evaluation of patients with known operative dates revealed 6 CRSs before April 26, 2023 (among 75 patients, 8%) and 0 CRSs after that date (45 patients). This timeframe corresponds to the introduction of quilting suture. Pearson’s correlation test demonstrated a significant positive correlation between BMI and operative time (*r* = 0.309, *p* < 0.001). No significant correlations were identified between BMI and defect size (*p* = 0.177) or between defect size and operative time (*p* = 0.275) (Table 4). Contingency analyses revealed statistically significant associations between BMI category and postoperative complications (χ² = 10.5, *p* = 0.004), as well as between sex and complications (χ² = 9.68, *p* = 0.002) (Table 5). Complete QoL score data were available for 104 patients. Patient-reported outcomes demonstrated substantial improvement across all domains. EuraHS-QoL total scores decreased from 57 preoperatively to 0 at 12 months, with parallel improvements in pain, functional restriction, and cosmetic scores (Table 6; Fig. 6). Similarly, the total CCS score decreased from 35.5 preoperatively to 0 at 12 months, with consistent reductions in pain, movement limitations, and sensory complaints (Table 7; Fig. 7).

**Table 1:** Baseline characteristics and indications

Variables	Values
Patients, n	120
Sex, n (%)	
Female	96 (80%)
Male	24 (20%)
Age at surgery (years), median (range)	42 (23–82)
BMI (kg/m²), median (range)	23.0 (17.1–34.0)
Previous abdominal operation, n (%)	55 (45.8%)
Indication, n (%)	
Diastasis recti + umbilical hernia	83 (69.2%)
Incisional hernia	39 (32.5%)
Other primary ventral hernia	15 (12.5%)

**Table 2:** Operative details

Variables	Values
Operative time, median (range)	183 min (118–420)
TAR, n	8 (6.7%)
Mesh type, n	
Polypropylene	97 (80.8%)
Poly-4-hydroxybutyrate (P4HB)	23 (19.2%)
Drain 1 removal, days (range)	3 (1–21)
Drain 2 removal, days (range)	8 (4–24)

**Table 3:** Postoperative outcomes and follow-up

Follow-up / outcomes	Value
Length of stay (days), median (range)	3 (2–10)
Follow-up (months), median (range)	31 (12–81)
Follow-up > 60 months, n (%)	22 (18.3%)
Complications, n (%)	8 (6.7%)
Clavien-Dindo I	1 (0.8%)
Clavien-Dindo II	1 (0.8%)
Clavien-Dindo IIIa	5 (4.2%)
Clavien-Dindo IIIb	1 (0.8%)
Complications type, n (%)	
Seroma	6 (5%)
Hematoma	2 (1.6%)
Recurrence, n	0

**Table 4:** Pearson’s correlation test

	BMI	Hernia/DR width
**Hernia/DR width**	Pearsons’s *r*: 0.091 *p* = 0.177	-
**Operation time**	Pearsons’s *r*: 0.309 *p* < 0.001	Pearsons’s *r*: 0.056 *p* = 0.275

**Table 5:** Contingency analyses

	Complications		
	No	Yes	
**BMI (***n* = 106)			*p* = 0.004
**< 25**	76 (71.7%)	2 (1.9%)	
**≥** **25**	22 (20.8%)	6 (5.7%)	
**Sex (***n* = 120)			*p* = 0.002
**F**	93 (77.5%)	3 (2.5%)	
**M**	19 (15.8%)	5 (4.2%)	

**Table 6:** EuraHS-QoL analisys (*n* = 104)

	Pre-operative	1 month	3 months	12 months
Total score	57 (48–66)	45 (32–51) *p* = 0.0031	6 (1–10) *p* < 0.01	0 (0–6) *p* < 0.01
Pain	17 (13–22)	16 (8–24) *p* = 0.420	0 (0–7) *p* < 0.01	0 (0–3) *p* < 0.01
Restriction	26 (18–33)	27 (22–33) *p* = 0.680	6 (1–10) *p* < 0.01	0 (0–2) *p* < 0.01
Cosmetic	14 (10–18)	1 (0–4) *p* < 0.01	0 (0–4) *p* < 0.01	0 (0–3) *p* < 0.01

**Table 7:** Carolinas Comfort Scale analisys (*n* = 104)

	Pre-operative	1 month	3 months	12 months
Total score	35.5 (28–43)	30 (19–38) *p* < 0.01	4 (0–8) *p* < 0.01	0 (0–4) *p* < 0.01
Pain	20 (14–27)	14 (9–18) *p* < 0.01	2 (0–4) *p* < 0.01	0 (0–2) *p* < 0.01
Movement	15 (10–21)	12 (7–18) *p* < 0.01	2 (0–4) *p* < 0.01	0 (0–4) *p* < 0.01
sensation	n.a.	4 (0–8) p = n.a.	0 (0–2) p = n.a.	0 (0–2) p = n.a.

## Discussion

Abdominal wall reconstruction (AWR) in minimally invasive surgery (MIS) has undergone substantial evolution over the past decade, reflecting a more sophisticated understanding of abdominal wall biomechanics, the functional implications of DR, and the limitations of traditional laparoscopic and open ventral hernia repairs [3, 15, 23, 29, 30]. The traditional laparoscopic techniques, IPOM and IPOM-plus, have long represented the standard minimally invasive approach for ventral hernia repair, but their reliance on intraperitoneal mesh placement exposes patients to well-recognized risks, such as adhesions, chronic pain, and visceral injury [7]. These limitations have driven the development of new extraperitoneal techniques designed to maintain the benefits of laparoscopy while avoiding peritoneal entry and enabling sublay mesh placement in a more favorable biological plane [23, 31, 32].

As recent guidelines and systematic reviews emphasize, current AWR can no longer be reduced to a simplistic dichotomy between open and laparoscopic approaches; instead, the key determinants of safety and durability are the access pathway, the working space, and the final mesh plane [3, 29]. In a previous article, we discussed the pragmatic classification of the newest MIS AWR techniques by access orientation: anterior vs. posterior. Within posterior MIS AWR, a further clinically meaningful subdivision is intraperitoneal versus extraperitoneal repair [32]. We have provided an updated classification of modern MIS AWR techniques described in the literature in **Supplementary S****1**.

This study provides the largest available dataset on TESAR, demonstrating that this anterior extraperitoneal technique, unique in reaching the retromuscular sublay plane from anterior access, appears to achieve outcomes that are in line with those reported for posterior extraperitoneal techniques in the literature, although no direct comparison can be made, while preserving the ergonomic and visceral safety advantages of anterior endoscopy [23]. This approach, introduced for the first time by our group, has already been described for the treatment of ventral and incisional hernias in a relatively small series of patients at the beginning of our experience [20]. The present study included 120 patients, with a median BMI of 23 and prevalence of combined DR and umbilical hernia (69.2%) (Table 3). This case mix reflects the typical population affected by symptomatic DR, which disproportionately impacts women in the postpartum period [23, 33]. The operative time (median of 183 min) aligns with ranges reported for endoscopic extraperitoneal retromuscular repairs such as TES, TEA, or e-Rives (102–285 min) [10, 31, 34] and is comparable with prior TESAR experiences published by our group [20, 23]. Complete, partial or unilateral TAR was necessary in only 6.7% of patients, confirming that TESAR can accommodate moderate-to-large defects with a setting compatible even with advanced dissections, using accessory trocars if needed.

The overall postoperative morbidity rate was 6.7%, which included seroma (5%) and hematoma/bleeding (1.6%). No SSIs were registered. These results are favorable when viewed in the context of current literature. Anterior onlay repair techniques (i.e., SCOLA/REPA/EPAR) report high seroma rates (12–27%) and occasional skin ischemia, which are directly linked to the biology of the preaponeurotic dead space [16, 18, 35, 36]. In our series TESAR was associated with a low seroma incidence, especially after the introduction of quilting suture. Posterior extraperitoneal sublay repair techniques (i.e., eTEP, TES, TEA, eTPA) report seroma rates ranging from 0.8 to 7.1% and recurrence rates ranging from 0 to 2.7%, but with limited follow-up [10, 31, 32, 34, 37, 38], and the TESAR complication rate aligns closely with these approaches. A particularly noteworthy finding is the absence of clinically relevant seromas after the introduction of quilting suture (from 8% to 0%). This finding is consistent with previous reports, especially from the plastic surgery literature, suggesting that seroma formation in anterior plane surgery may be, at least in part, reducible [39–44]. However, in our series this observation derives from an unadjusted before-and-after comparison and may be influenced by temporal factors such as surgical learning curve and changes in perioperative management. Postoperative complications demonstrated meaningful associations with patient-related and procedural variables. BMI emerged as a significant determinant: patients with a higher BMI had a greater likelihood of experiencing complications, which is consistent with the statistically significant association identified in the contingency analysis between BMI categories and postoperative complications (χ² = 10.5, *p* = 0.004). Operative time was also positively correlated with BMI (*r* = 0.309, *p* < 0.001), suggesting that increased adiposity not only prolongs the technical demands of the procedure but also may indirectly increase morbidity risk. In terms of sex, complications were more common among male patients, with a significant association documented between postoperative events and sex distribution (χ² = 9.68, *p* = 0.002). Although males constituted only 20% of the cohort, they were disproportionately represented among higher Clavien–Dindo grades, a finding that parallels prior evidence showing sex-related differences in DR morphology and tissue quality. Together, these correlations underscore the importance of careful preoperative risk stratification, particularly in patients with elevated BMIs or male sex, and support the role of the TESAR as a technique that remains safe across variable patient profiles, provided that patient-specific anatomic and functional considerations are appropriately integrated into surgical planning.

In terms of recurrence rate, this study revealed the absence of recurrences, even in the group with extended follow-up (18% of patients), which should be interpreted more as hypothesis-generating rather than definitive evidence of long-term superiority. The best results for posterior MIS extraperitoneal repairs reported in the literature (eTEP, TES, and TEA) include recurrence rates of 0–2.7%, with substantially shorter follow-up (6–18 months) [31, 32, 34, 37]. According to these data, the long-term durability of TESAR may be biologically plausible because it reproduces the proven principles of retromuscular sublay repair, with wide overlap, a vascularized mesh bed, isolation from the subcutaneous dead space, and extraperitoneal mesh location, reducing late mesh-related complications [21]. From a technical and biological standpoint, TESAR enables true “end-to-end” reconstruction of the linea alba in DR repair, allowing healing through direct collagen bridges between opposing fascial edges. Conversely, plication-based techniques incorporate redundant midline tissue within the repair, resulting in a non–end-to-end scar that heals mainly by fibrinous adhesion along the suture tracks. This mechanism leads to delayed maturation and reduced mechanical strength, with prolonged load sharing on the sutures—an effect that becomes more pronounced as diastasis width increases. These biological and biomechanical differences provide a plausible explanation for the durability observed with TESAR in long-term follow-up. By eliminating interposed tissue, TESAR creates a thin, compact scar with immediate tissue-to-mesh contact, promoting faster collagen maturation and good biomechanical stability.

The QoL and functional recovery results were encouraging as well. The EuraHS-QoL scores improved dramatically in all the domains: the total score significantly decreased from 57 to 0 at 12 months, the pain and restraint scores achieved near-complete resolution, and the cosmetic domain score decreased from 14 to 0 (*p* < 0.01). Similarly, the CCS scores reached 0 across all the domains at 12 months (Tables 6 and 7; Figs. 6 and 7). These improvements mirror findings from prior work demonstrating that abdominal wall reconstruction improves functional stability, posture, and aesthetics well-being [23, 33].

The marked improvement in patient-reported outcomes observed in this series should be interpreted considering the growing evidence that DR itself is a symptomatic condition. As consistently demonstrated by Olsson et al., surgical repair of DR, regardless of the specific technique adopted, results in significant improvements in core function, pain, and QoL. Therefore, the symptomatic benefit observed in our cohort may be attributed primarily to the restoration of the linea alba and the integrity of the abdominal wall rather than to the TESAR technique itself. The role of the surgical approach lies instead in determining durability, biomechanical strength, complication profile, and long-term outcomes [33, 45]. In our group, all posterior approaches were used, depending on the indications. Compared with other techniques, posterior extraperitoneal approaches offer several advantages: no preaponeurotic dead space, low seroma incidence, and excellent biomechanics. However, they also present well-documented limitations: first, they can be more easily performed with a robotic platform, and they have steep learning curves, especially for crossover maneuvers, retrorectus bleeding and hematoma risk, peritoneal ruptures leading to loss of workspace, and greater technical demand in healed or reoperative abdomens [10, 34, 37]. In the case of PeTEP, a valid and widespread technique, preperitoneal dissection may present difficulties linked to a very thin peritoneum, with the possibility of injury to the posterior plane and the need to modify the strategy. Furthermore, the posterior plane is very weak compared with the retromuscular plane, and this finding must be evaluated with further studies in the case of incisional defect repair [12].

TESAR avoids these pitfalls through a “familiar” anterior field, allowing easy access to the linea alba and controlled entry into the retrorectus space. For patients with multiple previous laparotomies or a history of intra-abdominal sepsis, in the absence of intestinal transit problems, TESAR is particularly appealing because its extraperitoneal, anterior-retromuscular approach allows for abdominal wall reconstruction without breaching the peritoneal cavity. This is especially valuable in the so-called “hostile abdomen”, where dense visceral adhesions are common: by avoiding peritoneal entry, TESAR can spare the surgeon from hazard and not necessarily adhesiolysis, thereby reducing the risk of inadvertent bowel injury, missed enterotomy, postoperative sepsis, and all the downstream consequences that may follow an intestinal lesion. In this setting, the main advantage of TESAR, which is that it is outside the abdominal cavity and performs Rives-Stoppa repair, is a genuine safety benefit.

Another relevant finding is the absence of postoperative bulging, a complication whose pathogenesis remains debated and that has been described for other techniques [10, 46, 47]. Proposed mechanisms include altered biomechanical muscle adaptation, possible iatrogenic injury to medially coursing neurovascular bundles in the upper abdomen, and transversus muscle overload due to crossover maneuvers without posterior layer reconstruction, which is not always feasible [48, 49]. In our series, the lack of bulging may be explained by the preservation of effective midline tension between the transversus muscles, which, when clinically indicated, can be further reinforced by medial plication of the posterior layer.

Although no direct comparison can be made with the anterior approaches, mainly belonging to the ENDOR family, the TESAR may provide similar outcomes with the other methods in terms of seroma rate, recurrence rate and biomechanics because onlay mesh placement, bridge mesh and simple plication remain biologically inferior to sublay reinforcement [21, 22]. TESAR achieves true sublay repair and avoids the inherent weaknesses of the onlay plane.

The other group of stapler-based plications offers an interesting alternative with good results, standardized midline tightening and a reduced operating time. However, since it is a section/suture of both planes, limitations remain, according to the indications (currently limited to smaller defects and DR < 5 cm), the risk of excessive tension of the posterior layer, which sometimes requires a release of the transversus fascia, and bleeding rates of 2.7–3.6% [32, 50–52].

TESAR avoids these constraints with direct, controlled suture-based linea alba reconstruction and a more physiological restoration of midline tension vectors, combining advantages traditionally segregated between anterior and posterior approaches. In summary, TESAR appears to provide a balanced combination of the durability of posterior sublay repairs, the safety of anterior access, and the cosmetic advantages of preaponeurotic dissection. This overlooked aspect, related to concepts specific to plastic surgery, derives from the favorable degree of skin retraction that an anterior approach allows, in contrast to a posterior approach in which there is no dissection of the prefascial space. This dissection determines the redistribution and retraction of the skin, which, especially in thin patients, may be associated with favorable cosmetic outcomes.

This study has several limitations. The heterogeneity of the study population, including patients with DR, primary ventral hernias, and incisional hernias, represents an important factor when interpreting outcomes. These conditions differ in physiopathology, defect characteristics, and surgical objectives, which may influence both recurrence risk and patient-reported outcomes. The retrospective, observational, non-comparative design restricts direct conclusions against other minimally invasive techniques, and although the results were contextualized using published series, differences in patient selection, defect complexity, and reporting standards across studies reduce the strength of cross-study comparisons [32]. Our outcomes may reflect advanced team expertise and reproducibility in lower-volume settings, where learning-curve effects have been shown to affect complication profiles in techniques such as eTEP [10]. While follow-up was long (up to 81 months, median 31 months, 18.3% exceeding 60 months), not all patients completed standardized long-term evaluation. Recurrence assessment was not standardized, as imaging was not performed in all patients; although the clinical evaluation during follow up was carried out by expert surgeons, asymptomatic or subclinical recurrences cannot be excluded. No specific data has been analyzed which could indicate a greater risk of recurrence in specific subgroups, therefore the heterogeneity of patients should be considered in the future to better define the indications.

The cohort’s relatively low BMI and limited need for TAR restrict generalizability to morbidly obese patients or those with very large defects, whose outcomes may differ substantially [22, 23, 53]. Finally, although quilting suture eliminated seroma in later cases, broader validation is needed, since seroma remains a hallmark complication of anterior approaches [16, 18, 35, 36].

Further research would benefit from multicenter trials comparing TESAR to other techniques, biomechanical imaging studies assessing the restoration of abdominal wall function and the evaluation of robotic TESAR, which may enhance ergonomics in retrorectus dissection.

In conclusion, TESAR appears to be a safe and effective minimally invasive technique for selected patients, with encouraging functional and long-term outcomes. In this single-center retrospective series, the technique was associated with low morbidity and marked improvement in patient-reported outcomes, suggesting that TESAR may represent a promising minimally invasive option for treating DR and primary and incisional midline ventral hernias, particularly in patients with prior abdominal operations, or a high risk of visceral adhesions. However, the absence of a comparator group, the heterogeneous population, and non-standardized recurrence assessment warrant cautious interpretation. Further prospective, multicenter, and comparative studies are required to better define its role within current abdominal wall reconstruction.

## Supplementary Information

Below is the link to the electronic supplementary material.

Supplementary Material 1

Supplementary Material 2

Supplementary Material 3

## References

[1] Muysoms FE, Miserez M, Berrevoet F, Campanelli G, Champault GG, Chelala E et al (2009) Classification of primary and incisional abdominal wall hernias. Hernia 13:407–41419495920 10.1007/s10029-009-0518-xPMC2719726

[2] Hernández-Granados P, Henriksen NA, Berrevoet F, Cuccurullo D, López-Cano M, Nienhuijs S et al (2021) European Hernia Society guidelines on management of rectus diastasis. Br J Surg 108:1189–119134595502 10.1093/bjs/znab128PMC10364860

[3] Silecchia G, Campanile FC, Sanchez L, Ceccarelli G, Antinori A, Ansaloni L et al (2015) Laparoscopic ventral/incisional hernia repair: updated guidelines from the EAES and EHS endorsed Consensus Development Conference. Surg Endosc 29:2463–248426139480 10.1007/s00464-015-4293-8

[4] Huang X, Shao X, Cheng T, Li J (2024) Laparoscopic intraperitoneal onlay mesh (IPOM) with fascial repair (IPOM–plus) for ventral and incisional hernia: a systematic review and meta–analysis. Hernia 28:385–40038319440 10.1007/s10029-024-02983-4

[5] Robinson TN, Clarke JH, Schoen J, Walsh MD (2005) Major mesh–related complications following hernia repair. Surg Endosc 19:1556–156016211441 10.1007/s00464-005-0120-y

[6] Brill JB, Turner PL (2011) Long–term outcomes with transfascial sutures versus tacks in laparoscopic ventral hernia repair: a review. Am Surg 77:458–465 21679556

[7] Maskal SM, Ellis RC, Mali O, Lau B, Messer N, Zheng X et al (2024) Long–term mesh–related complications from minimally invasive intraperitoneal onlay mesh for small to medium–sized ventral hernias. Surg Endosc 38:2019–202638424284 10.1007/s00464-024-10716-yPMC10978620

[8] Aliseda D, Sanchez–Justicia C, Zozaya G, Lujan J, Almeida A, Blanco N et al (2022) Short–term outcomes of minimally invasive retromuscular ventral hernia repair using an enhanced view totally extraperitoneal approach: systematic review and meta–analysis. Hernia 26:1511–152035044545 10.1007/s10029-021-02557-8PMC9684241

[9] Belyansky I, Daes J, Radu VG, Balasubramanian R, Zahiri HR, Weltz AS et al (2017) A novel approach using the enhanced–view totally extraperitoneal technique for laparoscopic retromuscular hernia repair. Surg Endosc 32:1525–153228916960 10.1007/s00464-017-5840-2

[10] Mazzola P, de Figueiredo S, Belyansky I, Lu R (2022) Pitfalls and complications of enhanced–view totally extraperitoneal approach to abdominal wall reconstruction. Surg Endosc 37:3354–336336575221 10.1007/s00464-022-09843-1

[11] Mitura K, Romańczuk M, Kisielewski K, Mitura B (2022) eTEP–RS for incisional hernias in a non–robotic center. Is laparoscopy enough to perform a durable MIS repair of the abdominal wall defect? Surg Endosc 37:1392–140035680666 10.1007/s00464-022-09365-wPMC9181889

[12] Alpuche HAV, Torres FR, González JPS (2024) Early results of eTEP access surgery with preperitoneal repair of primary midline ventral hernias and diastasis recti: a 33–patient case series of PeTEP. Surg Endosc 38:3204–321138637338 10.1007/s00464-024-10832-9

[13] Malcher F, Lima DL, Lima RNCL, Cavazzola LT, Claus CMP, Dong CT et al (2021) Endoscopic onlay repair for ventral hernia and rectus abdominis diastasis repair: why so many different names for the same procedure? A qualitative systematic review. Surg Endosc 35:5414–542134031740 10.1007/s00464-021-08560-5

[14] Gómez–Menchero J, Guadalajara Jurado JF, Suárez Grau JM, Bellido Luque JA, García Moreno JL, Alarcón del Agua I et al (2018) Laparoscopic intracorporeal rectus aponeuroplasty (LIRA technique): a step forward in minimally invasive abdominal wall reconstruction for ventral hernia repair. Surg Endosc 32:3502–350829344785 10.1007/s00464-018-6070-y

[15] Bellido Luque J, Bellido Luque A, Valdivia J, Suarez Grau JM, Gomez Menchero J, García Moreno J et al (2014) Totally endoscopic surgery on diastasis recti associated with midline hernias. The advantages of a minimally invasive approach. Prospective cohort study. Hernia 19:493–50125142493 10.1007/s10029-014-1300-2

[16] Claus CMP, Malcher F, Cavazzola LT, Furtado M, Morrell A, Azevedo M et al (2018) Subcutaneous onlay laparoscopic approach (SCOLA) for ventral hernia and rectus abdominis diastasis repair: technical description and initial results. Arq Bras Cir Dig 31:e139930539974 10.1590/0102-672020180001e1399PMC6284377

[17] Dong CT, Sreeramoju P, Pechman DM, Weithorn D, Camacho D, Malcher F (2020) Subcutaneous onlay endoscopic approach mesh repair for small midline ventral hernias with diastasis recti: an initial US experience. Surg Endosc 35:6449–645433206243 10.1007/s00464-020-08134-x

[18] Juárez Muas DM (2018) Preaponeurotic endoscopic repair of diastasis recti associated or not to midline hernias. Surg Endosc 33:1777–178230229321 10.1007/s00464-018-6450-3

[19] Gandhi JA, Shinde P, Kothari B, Churiwala JJ, Banker A (2020) Endoscopic pre–aponeurotic repair technique with meshplasty for treatment of ventral hernia and rectus abdominis diastasis. Indian J Surg 86:339–343

[20] Fiori F, Ferrara F, Gentile D, Gobatti D, Stella M (2019) Totally endoscopic sublay anterior repair for ventral and incisional hernias. J Laparoendosc Adv Surg Tech A 29:505–513 10.1089/lap.2018.0807 30807248

[21] Timmermans L, de Goede B, van Dijk SM, Kleinrensink GJ, Jeekel J, Lange JF (2014) Meta–analysis of sublay versus onlay mesh repair in incisional hernia surgery. Am J Surg 207:980–98824315379 10.1016/j.amjsurg.2013.08.030

[22] Shah DK, Patel SJ, Chaudhary SR, Desai NR (2023) Comparative study of onlay versus sublay mesh repair in the management of ventral hernias. Updates Surg 75:1991–199637195549 10.1007/s13304-023-01532-5

[23] Fiori F, Ferrara F, Gobatti D, Gentile D, Stella M (2020) Surgical treatment of diastasis recti: the importance of an overall view of the problem. Hernia 25:871–88232564225 10.1007/s10029-020-02252-0

[24] Papanikolaou A, Minger E, Pais MA, Constantinescu M, Olariu R, Grobbelaar A et al (2022) Management of postoperative seroma: recommendations based on a 12–year retrospective study. J Clin Med 11:506236078992 10.3390/jcm11175062PMC9457167

[25] Clavien PA, Barkun J, de Oliveira ML, Vauthey JN, Dindo D, Schulick RD et al (2009) The Clavien–Dindo classification of surgical complications: five–year experience. Ann Surg 250:187–19619638912 10.1097/SLA.0b013e3181b13ca2

[26] Muysoms F, Campanelli G, Champault GG, DeBeaux AC, Dietz UA, Jeekel J et al (2012) EuraHS: the development of an international online platform for registration and outcome measurement of ventral abdominal wall hernia repair. Hernia 16:239–25022527930 10.1007/s10029-012-0912-7PMC3360853

[27] Heniford BT, Lincourt AE, Walters AL, Colavita PD, Belyansky I, Kercher KW et al (2018) Carolinas Comfort Scale as a measure of hernia repair quality of life: a reappraisal utilizing 3788 international patients. Ann Surg 267:171–17627655239 10.1097/SLA.0000000000002027

[28] Pollock H, Pollock T (2000) Progressive tension sutures: a technique to reduce local complications in abdominoplasty. Plast Reconstr Surg 105:2583–258610845315 10.1097/00006534-200006000-00047

[29] Bittner R, Bain K, Bansal VK, Berrevoet F, Bingener J, Chen D et al (2019) Update of guidelines for laparoscopic treatment of ventral and incisional abdominal wall hernias (International Endohernia Society)—part B. Surg Endosc 33:3511–354931292742 10.1007/s00464-019-06908-6PMC6795640

[30] Bittner R, Bain K, Bansal VK, Berrevoet F, Bingener J, Chen D et al (2019) Update of guidelines for laparoscopic treatment of ventral and incisional abdominal wall hernias (International Endohernia Society)—part A. Surg Endosc 33:3069–313931250243 10.1007/s00464-019-06907-7PMC6722153

[31] Li B, Qin C, Bittner R (2018) Totally endoscopic sublay (TES) repair for midline ventral hernia: surgical technique and preliminary results. Surg Endosc 34:1543–155030374792 10.1007/s00464-018-6568-3

[32] Ferrara F, Fiori F (2024) Laparoendoscopic extraperitoneal surgical techniques for ventral hernias and diastasis recti repair: a systematic review. Hernia 28:2111–212439312025 10.1007/s10029-024-03144-3PMC11530491

[33] Olsson A, Kiwanuka O, Wilhelmsson S, Sandblom G, Stackelberg O (2019) Cohort study of the effect of surgical repair of symptomatic diastasis recti abdominis on abdominal trunk function and quality of life. BJS Open 3:750–75831832581 10.1002/bjs5.50213PMC6887686

[34] Li B, Qin C, Bittner R (2020) Endoscopic totally extraperitoneal approach technique for primary ventral hernia repair. Surg Endosc 34:3734–374132342218 10.1007/s00464-020-07575-8PMC7326894

[35] Kler A, Wilson P (2020) Total endoscopic–assisted linea alba reconstruction for treatment of umbilical/paraumbilical hernia and rectus abdominis diastasis is associated with unacceptable persistent seroma formation: a single–centre experience. Hernia 24:1379–138532691174 10.1007/s10029-020-02266-8

[36] Bellido–Luque J, Bellido–Luque A, Valdivia J, Suárez–Grau JM, Gómez–Menchero J, García–Moreno JL et al (2022) Severe rectus diastasis with associated midline hernia in males: high recurrence in mid–term follow–up of minimally invasive surgical technique. Hernia 27:335–34536454301 10.1007/s10029-022-02706-7

[37] Moga D, Buia F, Oprea V (2021) Laparo–endoscopic repair of ventral hernia and rectus diastasis. JSLS 25(2):e2020.00103. 10.4293/JSLS.2020.0010310.4293/JSLS.2020.00103PMC808833433981136

[38] Li B, Qin C, Liu D, Miao J, Yu J, Bittner R (2021) Subxiphoid top–down endoscopic totally preperitoneal approach for midline ventral hernia repair. Langenbecks Arch Surg 406:2125–213234297175 10.1007/s00423-021-02259-w

[39] Baroudi R, Ferreira C (1998) Seroma: how to avoid it and how to treat it. Aesthet Surg J 18:439–44119328174 10.1016/s1090-820x(98)70073-1

[40] Pollock T, Pollock H (2013) Progressive tension sutures in abdominoplasty. Body contouring and liposuction. Elsevier, Philadelphia, pp 336–344

[41] Sforza M, Husein R, Andjelkov K, Rozental–Fernandes PC, Zaccheddu R, Jovanovic M (2015) Use of quilting sutures during abdominoplasty to prevent seroma formation: are they really effective? Aesthet Surg J 35:574–58025953479 10.1093/asj/sju103

[42] Martins MRC, Nahas FX, Gomes HC, Ferreira LM, Mendes JA, Juliano Y (2022) The effect of quilting sutures on the tension required to advance the abdominal flap in abdominoplasty. Aesthet Surg J 42:628–63434791039 10.1093/asj/sjab395

[43] Nahas FX, Ferreira LM, Ghelfond C (2007) Does quilting suture prevent seroma in abdominoplasty? Plast Reconstr Surg 119:1060–106417312514 10.1097/01.prs.0000242493.11655.68

[44] Pollock TA, Pollock H (2012) Progressive tension sutures in abdominoplasty: a review of 597 consecutive cases. Aesthet Surg J 32:729–74222751080 10.1177/1090820X12452294

[45] Olsson A, Kiwanuka O, Sandblom G, Stackelberg O (2021) Evaluation of functional outcomes following rectus diastasis repair: an up–to–date literature review. Hernia 25:905–91434302558 10.1007/s10029-021-02462-0PMC8370918

[46] Daes J, Hanssen A, Luque E, Rocha J (2025) Abdominal wall contour and muscle changes after eTEP repair for small ventral hernias and diastasis: a quality improvement study. Surg Endosc 39:4365–437540442354 10.1007/s00464-025-11816-z

[47] Halpern D, Panahi A, Cordero K, Pan J, Pacheco TBS, Joutovsky B (2026) Effect of posterior rectus sheath closure on outcomes of enhanced total extraperitoneal ventral hernia repair. Hernia 30:1–10 10.1007/s10029-026-03631-9 41790287

[48] Manetti G, Lolli MG, Belloni E, Nigri G (2021) A new minimally invasive technique for the repair of diastasis recti: a pilot study. Surg Endosc 35:4028–403433661384 10.1007/s00464-021-08393-2PMC8195785

[49] Nahabedian MY (2018) Management strategies for diastasis recti. Semin Plast Surg 32:147–15430046291 10.1055/s-0038-1661380PMC6057788

[50] Carrara A, Lauro E, Fabris L, Frisini M, Rizzo S (2019) Endo–laparoscopic reconstruction of the abdominal wall midline with linear stapler: the THT technique. Early results of the first case series. Ann Med Surg 38:1–7 10.1016/j.amsu.2018.12.002 PMC6302139 30581569

[51] Carrara A, Lauro E, Fabris L, Frisini M, Rizzo S, Biondi A et al (2020) Prospective observational study of abdominal wall reconstruction with THT technique in primary midline defects with diastasis recti: clinical and functional outcomes in 110 consecutive patients. Surg Endosc 35:5104–511432964305 10.1007/s00464-020-07997-4

[52] Carrara A, Costa TN, Nava FL, Fabris L, Zuolo M, Dorna AE et al (2021) Trentino Hernia Team Technique plus endoscopic transversus abdominis release for large ventral incisional hernias: description of the first case. Videoscopy 31(3). 10.1089/vor.2020.0658

[53] Brito ÍM, Meireles R, Baltazar J, Brandão C, Sanches F, Freire–Santos MJ (2020) Abdominoplasty and patient safety: the impact of body mass index and bariatric surgery on complication profile. Aesthet Plast Surg 44:1615–1624 10.1007/s00266-020-01725-y 32342171

