# Supplementary material for: Totally Endoscopic Sublay Anterior Repair (TESAR) for diastasis recti, primary and incisional ventral hernias: Long-term clinical outcomes and quality of life
Source: Hernia. 2026 Jul 31;30(1):305. doi: 10.1007/s10029-026-03808-2 (PMC13427856; doi:10.1007/s10029-026-03808-2)
Supplement: Supplementary file 2 — Supplementary Material 2 [file 10029_2026_3808_MOESM2_ESM.docx]

**Supplementary S1**

**Technical synopsis and classification of minimally invasive techniques**

This Supplementary file provides a concise technical glossary and a pragmatic classification of the most common abdominal wall minimally invasive techniques. Techniques are grouped by access route (posterior transperitoneal, posterior extraperitoneal, anterior extraperitoneal), principal working space, and definitive mesh plane. The aim is descriptive and focuses on reproducible operative concepts and the most characteristic complication vectors[1-4].

**IPOM (Intraperitoneal Onlay Mesh).** Laparoscopic intraperitoneal repair with a composite/anti-adhesive prosthesis placed inside the peritoneal cavity to cover the defect with overlap, typically fixed with sutures and/or tacks. Defect bridging is common; bulging and fixation-related pain can occur, and the intraperitoneal mesh position entails mesh-viscera interface considerations[5].

**IPOM-plus.** IPOM variant adding primary fascial closure of the defect (often with running or barbed suture) before intraperitoneal mesh placement. Intended to restore midline tension vectors and reduce bulging/recurrence versus bridging IPOM, while maintaining peritoneal entry, possible adhesiolysis, and an intraperitoneal prosthesis[6].

**Ventral TAPP (Transabdominal Preperitoneal ventral hernia repair).** Transperitoneal approach in which a peritoneal flap is created to develop the preperitoneal plane, the sac is reduced, the defect is closed, and a mesh is placed preperitoneally with wide overlap. The peritoneal flap is then closed to separate the mesh from viscera; durable flap creation and closure are key determinants[7].

**LIRA (Laparoscopic Intracorporeal Rectus Aponeuroplasty).** Intracorporeal midline reconstruction strategy performed transperitoneally, based on posterior rectus sheath/aponeurotic medial plication to recreate the linea alba followed by prosthetic reinforcement. It emphasizes tension redistribution and anatomic restoration, inherently requires peritoneal entry and advanced suturing. **Preperitoneal LIRA, PeIRA.** A variant of the LIRA that allows the preperitoneal positioning of the mesh[8-10].

**eTEP (enhanced-view Totally ExtraPeritoneal).** Posterior extraperitoneal retromuscular repair with a direct retromuscular approach, creating a large retrorectus workspace to allow linea alba reconstruction and wide retromuscular sublay mesh placement. eTEPas its peculiar technical aspect in the crossover, the step that allows the union of the three spaces, right and left retrorectus through the preperitoneal plane. Typical steps include retrorectus access, broad cranio-caudal dissection, crossover, repair of peritoneal breaches if needed, midline closure, and large mesh deployment with minimal fixation[11, 12].

**PeTEP (Preperitoneal extended Totally ExtraPeritoneal ).** A posterior extraperitoneal approach with a direct preperitoneal sovrapubic access performing a preperitoneal bottom up dissection, creating a large preperitoneal pocket for preperitoneal/sublay mesh positioning. Core determinants are maintaining pocket integrity managing peritoneal fragility/tears. **Cranial PeTEP** is a variant with a top-down dissection starting in the preperitoneal space at the sub-xiphoid area[13, 14].

**TES (Totally Endoscopic Sublay).** Totally endoscopic extraperitoneal sublay repair using retrorectus and/or preperitoneal planes to reduce the sac, close the defect, and place mesh in a true sublay position. Variations mainly involve access and plane-development sequence; the goal remains extraperitoneal sublay reinforcement with adequate overlap[15].

**TEA (Totally Extraperitoneal Approach).** Extraperitoneal preperitoneal repair characterized by a sovrapubic access, a bottom up extensive development of the midline extraperitoneal/preperitoneal plane, reduction of the hernia sac, defect closure, and placement of a large mesh in the preperitoneal position[16].

**eTPA (endoscopic Top-down Preperitoneal Approach).** A top-down preperitoneal variant (commonly subxiphoid entry) in which the preperitoneal plane behind the linea alba is developed caudally, the defect is closed, and a mesh is placed preperitoneally. This access can be sometimes advantageous for selected defects but is sensitive to peritoneal integrity[17].

**e-Rives (Endoscopic Rives-Stoppa concept).** Posterior endoscopic retromuscular repair with lateral retrorectus entry and bilateral retromuscular dissection, followed by reconstruction (posterior/anterior layer management as applicable) and retromuscular sublay mesh placement. Technical priorities include neurovascular preservation and meticulous retrorectus hemostasis[18].

**SIL-TES (Single-Incision Laparoscopic Totally Extraperitoneal Sublay).** Single-incision adaptation of extraperitoneal sublay repair, accessing and dissecting the retromuscular space through a single port-site incision, with defect closure and sublay mesh deployment. Benefits are fewer skin incisions; constraints are triangulation and ergonomics[19].

**MILOS (Mini- or Less-open Sublay Operation).** Hybrid mini/less-open technique with a small incision over the defect enabling transhernial entry and outward extraperitoneal/retrorectus dissection to achieve retromuscular sublay mesh reinforcement. Combines tactile control at the defect with extraperitoneal sublay principles[20].

**EMILOS (Endoscopic Mini/less-open Sublay).** Endoscopic-assisted evolution of MILOS using camera visualization to extend and control the retromuscular dissection and mesh placement while maintaining limited skin access[21, 22].

**E/MILOP (Endoscopic Mini/less-open Preperitoneal).** Mini/less-open transhernial technique preferentially developing the preperitoneal plane under endoscopic control, closing the defect, and placing mesh preperitoneally. The approach is attractive when a broad preperitoneal pocket can be safely maintained; it is sensitive to peritoneal injury in scarred abdomens[23].

**Stapled posterior plication techniques (miSAR; THT).** Posterior approaches using linear staplers to standardize midline tightening within the retromuscular compartment, followed by retromuscular mesh placement. Key limiting factors are posterior layer tension, the suture-section of the two sheats[24-27].

**ENDOR family** (generic term for endoscopic preaponeurotic onlay repairs as SCOLA, REPA, EPAR, SCOM, SVAWD,ELAR, MILAR, FESSA, TESLAR)**.** Anterior extraperitoneal endoscopic repairs performed in a preaponeurotic/subcutaneous working space with linea alba plication and sometimes an onlay mesh reinforcement. Multiple named variants share the same foundational concept; the dominant biological trade-off is creation of a large subq working space, driving seroma risk[4].

**SCOLA (Subcutaneous Onlay Laparoscopic Approach,** today **Subcutaneous Onlay Endoscopic Approach ).** Suprapubic endoscopic development of the preaponeurotic plane, exposure of the linea alba, midline plication, and sometimes an onlay polypropylene mesh placement in the subcutaneous compartment. In the first paper mesh was considered a surgeon's choice, today SCOLA is often referred to as a no-mesh operation[28, 29].

**REPA (Reparacion Endoscopica Pre-Aponeurotica).** Preaponeurotic endoscopic approach similar in concept to SCOLA, with midline reconstruction and onlay mesh placement. Technical differences largely relate to access and suturing sequence; seroma remains the signature complication vector[30].

**SVAWD (Subcutaneous Videosurgery for Abdominal Wall Defects).** Absolutely similar to REPA, with a sub coutaneous dissection bottom up, the plication of the fascia and a onlay large mesh[31].

**EPAR (Endoscopic Pre-Aponeurotic Repair).** Endoscopic preaponeurotic repair reconstructing the linea alba and reinforcing with onlay mesh. Shares the anterior corridor and dead-space biology of ENDOR proceduresgandhi[32].

**SCOM (Subcutaneous Onlay Mesh and related variants).** Anterior endoscopic onlay repairs sometimes using lateral access to develop the preaponeurotic plane, perform plication, and position an onlay mesh[33].

**ELAR (Endoscopic-assisted Linea Alba Reconstruction).** Anterior reconstruction technique that incises the anterior rectus sheaths plicating medially to recreate the linea alba, typically with mesh augmentation (onlay). Reconstructive intent is higher than simple plication, but seroma mitigation still depends on dead-space control[34].

**MILAR (Minimal Invasive Linea Alba Reconstruction).** The ELAR concept with a limited open acces. Minimally invasive linea alba reconstruction with limited access and preaponevrotic dissection, fascial incision an medial plication to restore midline anatomy, incorporating a biosynthetic/absorbable bridge-mesh[35].

**TESLAR (Total Endoscopic-assisted Linea Alba Reconstruction).** Endoscopic-assisted linea alba reconstruction variant with mesh reinforcement; reported experiences underline that wide preaponeurotic dissection can be associated with persistent seroma, emphasizing the need for systematic dead-space strategy (limited dissection, compression, quilting)[36].

**FESSA (Full Endoscopic Suprapubic Subcutaneous Access).** Anterior suprapubic full endoscopic subcutaneous access with bilateral anterior sheath incisions, exposure of rectus bellies, midline reconstruction, and onlay mesh anchored to sheath margins. Expands reconstructive steps within an anterior corridor; durability can be phenotype-sensitive in severe diastasis patterns[37].

**Bilayer endoscopic-assisted techniques.** Hybrid concept combining a limited open/periumbilical phase to approximate/close the umbilical defect and an endoscopic preaponevrotic phase for additional fascial suturing and reinforcement. Is usually a no-mesh repair[38].

**TESAR (Total Endoscopic Sublay Anterior Repair).** Anterior suprapubic endoscopic access with preaponeurotic dissection to fully expose the midline defect or diastasis, followed by controlled bilateral incision of the medial margin of anterior rectus sheaths to enter and develop the retrorectus plane. The posterior plane can be plicated to reload the transversus muscle if needed. A large mesh is deployed in the retromuscular (retrorectus) sublay position with wide overlap, and the anterior sheaths are closed to reconstruct the linea alba and compartmentalize the prosthesis. Because the anterior corridor creates a potential dead space, systematic subcutaneous-to-fascia quilting (progressive-tension) sutures are used to collapse this space and reduce seroma formation[39-41].

**Table A. Pragmatic classification of cited techniques by access route, working space, and definitive mesh plane.**

| **Technique / family** | **Access route** | **Principal working space** | **Definitive mesh plane** | **Midline closure / reconstruction** | **Typical niche and signature complication vector** |
| --- | --- | --- | --- | --- | --- |
| **IPOM** | Transperitoneal | Intraperitoneal | Intraperitoneal | Variable (often bridging) | Broad applicability; mesh-viscera interface, fixation-related pain/bulging |
| **IPOM-plus** | Transperitoneal | Intraperitoneal | Intraperitoneal | Yes (defect closure) | Improved bulging/recurrence vs bridging; intraperitoneal mesh risks remain |
| **Ventral TAPP** | Transperitoneal | Intraperitoneal + preperitoneal flap | Preperitoneal | Yes | Selected small-medium defects; adhesiolysis/enterotomy risk, peritoneal flap integrity |
| **LIRA/**  **Extrap LIRA-PeIRA** | Transperitoneal/  extraperitoneal | Intraperitoneal/ preperitoneal | Intraperitoneal/Peperitoneal | Yes | Biomechanics-oriented midline restoration; advanced intracorporeal suturing required |
| **eTEP** | Posterior extraperitoneal | Retrorectus (+/- crossover) | Retromuscular (retrorectus) | Yes | True sublay with low seroma; learning curve, bleeding, peritoneal tears/workspace loss |
| **PeTEP**  **Cranial PeTEP** | Posterior extraperitoneal | Preperitoneal bottom-up/Preperitoneal top-down | Preperitoneal | Yes | Wide preperitoneal pocket; peritoneal fragility and workspace loss with tears |
| **TES** | Posterior extraperitoneal | Retrorectus | Sublay (retrorectus) | Yes | Extraperitoneal sublay variants; plane integrity and ergonomics are key |
| **TEA** | Posterior extraperitoneal | Preperitoneal | Preperitoneal | Yes | Preperitoneal sublay; sensitive to peritoneal breaches |
| **eTPA** | Posterior extraperitoneal | Preperitoneal (top-down) | Preperitoneal | Yes | Cranial access for selected defects; peritoneal handling central |
| **e-Rives** | Posterior extraperitoneal | Retrorectus (lateral entry) | Retromuscular sublay | Yes | Rives-Stoppa endoscopic translation; bleeding risk and neurovascular preservation |
| **SIL-TES** | Posterior extraperitoneal | Retrorectus | Retromuscular sublay | Yes | Single-incision cosmetics; reduced triangulation/ergonomics |
| **MILOS** | Posterior extraperitoneal (hybrid) | Transhernial + retrorectus | Retromuscular sublay | Often yes | Mini-access extraperitoneal sublay; indication discipline for very wide defects |
| **EMILOS** | Posterior extraperitoneal (hybrid) | Transhernial + retrorectus (endoscopic-assisted) | Retromuscular sublay | Often yes | MILOS with enhanced visualization; similar constraints to MILOS |
| **E/MILOP** | Posterior extraperitoneal (hybrid) | Transhernial + preperitoneal | Preperitoneal sublay | Yes | Preperitoneal focus; peritoneal integrity in scarred abdomens |
| **Stapled posterior plication (THT/miSAR)** | Posterior extraperitoneal | Retrorectus | Retromuscular sublay | Yes (stapled tightening) | Standardized tightening; |
| **ENDOR family (SCOLA/REPA/EPAR/SCOM)** | Anterior extraperitoneal | Preaponeurotic / subcutaneous | Onlay , bridge, no-mesh | Yes (plication) | Low visceral risk; dead-space-driven seroma is signature issue |
| **SCOLA** | Anterior extraperitoneal | Preaponeurotic / subcutaneous | Onlay/no mesh | Yes | Diastasis +/- small hernia; seroma depends on dissection and dead-space control |
| **REPA** | Anterior extraperitoneal | Preaponeurotic / subcutaneous | Onlay | Yes | Similar to SCOLA; seroma mitigation is central |
| **SVAWD** | Anterior extraperitoneal | Preaponeurotic / subcutaneous | Onlay |  | Similar to REPA |
| **EPAR** | Anterior extraperitoneal | Preaponeurotic / subcutaneous | Onlay | Yes | Variant sequences; seroma remains dominant trade-off |
| **SCOM** | Anterior extraperitoneal | Preaponeurotic / subcutaneous | Onlay | Yes | Alternative access; dead space persists |
| **ELAR** | Anterior extraperitoneal | Preaponeurotic / subcutaneous | Onlay, bridge | Yes with bridge mesh | Reconstructive intent; seroma risk linked to dissection extent |
| **MILAR** | Anterior extraperitoneal | Preaponeurotic / subcutaneous | Onlay, bridge (sometimes biosynthetic) | Yes with bridge mesh | Material variation; dead-space strategy dictates seroma profile |
| **TESLAR** | Anterior extraperitoneal | Preaponeurotic / subcutaneous | Onlay | Yes | Reports of persistent seroma highlight dead-space biology |
| **FESSA** | Anterior extraperitoneal | Preaponeurotic / subcutaneous | Onlay, bridge | Yes with bridge mesh | Expanded anterior reconstruction; phenotype-sensitive recurrence in some cohorts |
| **Bilayer techniques** | Anterior (hybrid) | Limited open + preaponeurotic endoscopy | Usually no mesh | Yes | Balances control/invasiveness; outcomes depend on plane choice and dead-space management |
| **TESAR** | Anterior extraperitoneal (with retrorectus sublay) | Preaponeuroticretrorectus | Retromuscular (retrorectus) sublay | Yes | Extraperitoneal safety with true sublay; seroma minimized by systematic quilting |

**References**

1. Bittner R, Bain K, Bansal VK, Berrevoet F, Bingener‑Casey J, Chen D, et al (2019) Update of guidelines for laparoscopic treatment of ventral and incisional abdominal wall hernias (International Endohernia Society): part B. Surg Endosc 33:3511–3549
2. Bittner R, Bain K, Bansal VK, Berrevoet F, Bingener‑Casey J, Chen D, et al (2019) Update of guidelines for laparoscopic treatment of ventral and incisional abdominal wall hernias (International Endohernia Society): part A. Surg Endosc 33:3069–3139
3. Silecchia G, Campanile FC, Sanchez L, Ceccarelli G, Antinori A, Ansaloni L, et al (2015) Laparoscopic ventral/incisional hernia repair: updated guidelines from the EAES and EHS endorsed consensus development conference. Surg Endosc 29:2463–2484
4. Malcher F, Lima DL, Lima RNCL, Cavazzola LT, Claus C, Dong CT, et al (2021) Endoscopic onlay repair for ventral hernia and rectus abdominis diastasis repair: why so many different names for the same procedure? A qualitative systematic review. Surg Endosc 35:5414–5421
5. LeBlanc KA (2018) Laparoscopic incisional and ventral hernia repair. In: Management of abdominal hernias. Springer, Cham, pp 393–410
6. Huang X, Shao X, Cheng T, Li J (2024) Laparoscopic intraperitoneal onlay mesh with fascial repair (IPOM‑plus) for ventral and incisional hernia: a systematic review and meta‑analysis. Hernia 28:385–400
7. Maatouk M, Kbir GH, Mabrouk A, Rezgui B, Dhaou AB, Daldoul S, et al (2022) Can ventral TAPP achieve favorable outcomes in minimally invasive ventral hernia repair? A systematic review and meta‑analysis. Hernia 27:729–739
8. Gómez‑Menchero J, Guadalajara Jurado JF, Suárez Grau JM, Bellido Luque JA, García Moreno JL, Alarcón del Agua I, et al (2018) Laparoscopic intracorporeal rectus aponeuroplasty (LIRA technique): a step forward in minimally invasive abdominal wall reconstruction for ventral hernia repair. Surg Endosc 32:3502–3508
9. Gómez López JR, Navarro Morales L, Gómez Menchero J, Morales‑Conde S (2024) Preperitoneal LIRA: an alternative in primary midline hernia repair. Cir Esp (Engl Ed) 102:157
10. Gómez‑Menchero J, Lara Fernández Y, Morales‑Conde S, Balla A (2026) Preperitoneal endoscopic intracorporeal rectus aponeuroplasty (PeIRA) technique for midline ventral hernia repair: new technique based on LIRA concepts. Cir Esp (Engl Ed) 800305
11. Belyansky I, Daes J, Radu VG, Balasubramanian R, Reza Zahiri H, Weltz AS, et al (2017) A novel approach using the enhanced‑view totally extraperitoneal technique for laparoscopic retromuscular hernia repair. Surg Endosc 32:1525–1532
12. Daes J (2011) The enhanced‑view totally extraperitoneal technique for repair of inguinal hernia. Surg Endosc 26:1187–1189
13. Alpuche HAV, Torres FR, González JPS (2024) Early results of eTEP access surgery with preperitoneal repair of primary midline ventral hernias and diastasis recti: a 33‑patient case series of PeTEP. Surg Endosc 38:3204–3211
14. Muñoz‑Rodríguez JM, Román García de León L, Robin Valle de Lersundi Á, Blázquez‑Hernando LA, Medina Pedrique M, Fidalgo Martínez C, et al (2026) Preperitoneal enhanced‑view totally extraperitoneal technique in midline and lateral incisional hernia repair: early multicenter outcomes. Surgery 192:110041
15. Li B, Qin C, Bittner R (2018) Totally endoscopic sublay repair for midline ventral hernia: surgical technique and preliminary results. Surg Endosc 34:1543–1550
16. Li B, Qin C, Bittner R (2020) Endoscopic totally extraperitoneal approach technique for primary ventral hernia repair. Surg Endosc 34:3734–3741
17. Li B, Qin C, Liu D, Miao J, Yu J, Bittner R (2021) Subxiphoid top‑down endoscopic totally preperitoneal approach for midline ventral hernia repair. Langenbecks Arch Surg 406:2125–2132
18. Moga D, Buia F, Oprea V (2021) Laparo‑endoscopic repair of ventral hernia and rectus diastasis. JSLS 25:e2020.00103
19. Wang T, Tang R, Meng X, Zhang Y, Huang L, Zhang A, et al (2022) Comparative review of outcomes: single‑incision laparoscopic totally extraperitoneal sublay mesh repair versus laparoscopic intraperitoneal onlay mesh repair for ventral hernia. Updates Surg 74:1117–1127
20. Reinpold W, Schröder M, Berger C, Nehls J, Schröder A, Hukauf M, et al (2019) Mini‑ or less‑open sublay operation (MILOS): a new minimally invasive technique for the extraperitoneal mesh repair of incisional hernias. Ann Surg 269:748–755
21. Schwarz J, Reinpold W, Bittner R (2016) Endoscopic mini‑ or less‑open sublay technique (EMILOS): a new technique for ventral hernia repair. Langenbecks Arch Surg 402:173–180
22. De‑Carvalho JPV, Pivetta LGA, Amaral PHDF, Dias ERM, Macret JZ, Ribeiro HB, et al (2023) Endoscopic mini or less open sublay repair in ventral hernia correction: a minimally invasive technical alternative. Rev Col Bras Cir 50
23. Nakabayashi R, Matsubara T, Shimada G (2023) The endoscopic‑assisted or endoscopic mini‑ or less‑open preperitoneal approach for primary and incisional ventral hernia repair. Asian J Endosc Surg 16:482–488
24. Manetti G, Lolli MG, Belloni E, Nigri G (2021) A new minimally invasive technique for the repair of diastasis recti: a pilot study. Surg Endosc 35:4028–4034
25. Carrara A, Lauro E, Fabris L, Frisini M, Rizzo S (2019) Endo‑laparoscopic reconstruction of the abdominal wall midline with linear stapler: the THT technique. Early results of the first case series. Ann Med Surg 38:1–7
26. Carrara A, Catarci M, Fabris L, Zuolo M, Pellecchia L, Moscatelli P, et al (2020) Prospective observational study of abdominal wall reconstruction with THT technique in primary midline defects with diastasis recti: clinical and functional outcomes in 110 consecutive patients. Surg Endosc 35:5104–5114
27. Carrara A, Costa TN, Nava FL, Fabris L, Zuolo M, Dorna AE, et al (2021) Trentino Hernia Team Technique plus endoscopic transversus abdominis release for large ventral incisional hernias: description of the first case. Videoscopy 31:3
28. Dong CT, Sreeramoju P, Pechman DM, Weithorn D, Camacho D, Malcher F (2020) Subcutaneous onlay endoscopic approach mesh repair for small midline ventral hernias with diastasis recti: an initial US experience. Surg Endosc 35:6449–6454
29. Shinde PH, Chakravarthy V, Karvande R, Mahadik K, Gandhi J (2022) A novel modification of subcutaneous onlay endoscopic repair of midline ventral hernias with diastasis recti: an Indian experience. Cureus 14
30. Juárez Muas DM (2018) Preaponeurotic endoscopic repair of diastasis recti associated or not to midline hernias. Surg Endosc 33:1777–1782
31. Barchi LC, Franciss MY, Zilberstein B (2019) Subcutaneous videosurgery for abdominal wall defects: a prospective observational study. J Laparoendosc Adv Surg Tech A 29:523–530
32. Gandhi JA, Shinde P, Kothari B, Churiwala JJ, Banker A (2020) Endoscopic pre‑aponeurotic repair technique with meshplasty for treatment of ventral hernia and rectus abdominis diastasis. Indian J Surg 86:339–343
33. Makam R, Chamany T, Nagur B, Bilchod SS, Kulkarni A (2023) Laparoscopic subcutaneous onlay mesh repair for ventral hernia: our early experience. J Minim Access Surg 19:223–226
34. Köckerling F, Botsinis MD, Rohde C, Reinpold W, Schug‑Pass C (2017) Endoscopic‑assisted linea alba reconstruction. Eur Surg 49:71–75
35. Köhler G, Fischer I, Kaltenböck R, Schrittwieser R (2018) Minimal invasive linea alba reconstruction for the treatment of umbilical and epigastric hernias with coexisting rectus abdominis diastasis. J Laparoendosc Adv Surg Tech A 28:1223–1228
36. Kler A, Wilson P (2020) Total endoscopic‑assisted linea alba reconstruction for treatment of umbilical/paraumbilical hernia and rectus abdominis diastasis is associated with unacceptable persistent seroma formation: a single‑centre experience. Hernia 24:1379–1385
37. Bellido‑Luque J, Gómez‑Rosado JC, Bellido‑Luque A, Matamoros IS, Muñoz AN, Mompeán FO, et al (2022) Severe rectus diastasis with associated midline hernia in males: high recurrence in mid‑term follow‑up of minimally invasive surgical technique. Hernia 27:335–345
38. Ngo P, Cossa JP, Guéroult S, Pélissier E (2023) Minimally invasive bilayer suturing technique for the repair of concomitant ventral hernias and diastasis recti. Surg Endosc 37:5326–5334
39. Ferrara F, Fiori F (2024) Laparoendoscopic extraperitoneal surgical techniques for ventral hernias and diastasis recti repair: a systematic review. Hernia 28:2111–2124
40. Fiori F, Ferrara F, Gentile D, Gobatti D, Stella M (2019) Totally endoscopic sublay anterior repair for ventral and incisional hernias. J Laparoendosc Adv Surg Tech A 29:505–513
41. Fiori F, Ferrara F, Gobatti D, Gentile D, Stella M (2020) Surgical treatment of diastasis recti: the importance of an overall view of the problem. Hernia 25:871–882
