# Supplementary material for: Totally Endoscopic Sublay Anterior Repair (TESAR) for diastasis recti, primary and incisional ventral hernias: Long-term clinical outcomes and quality of life
Source: Hernia. 2026 Jul 31;30(1):305. doi: 10.1007/s10029-026-03808-2 (PMC13427856; doi:10.1007/s10029-026-03808-2)
Supplement: Supplementary file 1 — Supplementary Material 1 [file 10029_2026_3808_MOESM1_ESM.docx]

**Dear Editor,**

**We would like to sincerely thank the Reviewers for their thorough evaluation of our manuscript and for their insightful and constructive comments. We have carefully addressed all the concerns raised, which have significantly improved the clarity, rigor, and overall quality of the manuscript.**

**All changes have been incorporated into the revised version, and they are highlighted in the manuscript. Detailed point-by-point responses are provided below.**

**Reviewer #1**: The authors present a relatively large single-center experience with TESAR and long-term follow-up. The topic is clinically relevant given the growing interest in minimally invasive extraperitoneal abdominal wall reconstruction.
The abstract lacks important quantitative details. Please report the number of patients in each indication group, follow-up as median (IQR), the method used to assess recurrence, and the actual baseline and follow-up QoL scores. The conclusion should be more cautious and better reflect the observational design of the study.

We have comprehensively revised the abstract to include all requested quantitative details. Specifically, we have now reported the number of patients in each indication group, the follow-up expressed as median with interquartile range (IQR), the method used to assess recurrence, and the baseline and follow-up QoL values for both instruments. In addition, the conclusion has been revised to better reflect the observational and non-comparative design of the study.

The study population is heterogeneous, including patients with diastasis recti, primary ventral hernias, and incisional hernias. The authors should better define the primary indication for surgery and discuss the potential impact of this heterogeneity on the reported outcomes.
We agree with this important observation. We have clarified the primary surgical indication of TESAR (abdominal wall midline defects), stating that the majority of cases consisted of symptomatic diastasis recti associated with umbilical or other midline defects, followed by isolated primary and incisional hernias. Furthermore, we have expanded the discussion and limitations sections to explicitly address the heterogeneity of the population and its potential impact on outcomes, particularly with respect to recurrence risk and patient-reported outcomes.

Recurrence assessment was not standardized, as imaging was performed only when clinically indicated. This limitation should be acknowledged when interpreting the reported absence of recurrence.

We acknowledge this limitation. The manuscript has been revised to clearly state that recurrence assessment was not standardized and that imaging was not performed in all cases. This limitation has been explicitly discussed, and appropriate caution has been added when interpreting the absence of recurrence.
The discussion repeatedly compares TESAR favorably with other techniques despite the absence of a comparator group. Several statements should be toned down and the conclusions should better reflect the observational design of the study.

We fully agree and have extensively revised the discussion to avoid overinterpretation. All statements suggesting superiority or direct comparison with other techniques have been carefully rephrased. Comparisons with other approaches are now clearly contextualized as indirect and based on literature, explicitly acknowledging the absence of a comparator group.

The limitations section should more explicitly acknowledge the retrospective design, lack of a control group, potential selection bias, and non-standardized recurrence surveillance.

The limitations section has been substantially expanded to explicitly include the retrospective design, the absence of a control group, potential selection bias, the heterogeneity of the study population, non-standardized recurrence assessment, and the role of telemedicine in follow-up.

The conclusion that quilting sutures eliminated clinically relevant seromas should be interpreted cautiously, as this was an unadjusted before-and-after comparison potentially influenced by temporal and learning-curve effects.
We have revised this section to ensure a more cautious interpretation. The findings regarding quilting sutures are now clearly described as exploratory. We explicitly state that the analysis is unadjusted and may be influenced by temporal factors, including learning-curve effects and perioperative changes.

**Reviewer #2**: Congratulations on an excellent series of patients.  While you should be commended for these excellent outcomes, you must also understand the reader's speculation with reporting 0 infections and 0 recurrences.  Given your multiple methods of follow-up with supplemental virtual/telephone options, please explicitly add exactly how much follow-up was achieved by each method so that you can comment on this as a potential limitation in the discussion.

We thank the Reviewer for the positive feedback and thoughtful suggestions.

We have now explicitly quantified the follow-up modalities. The manuscript reports the proportion of patients assessed through in-person clinical examination versus telemedicine.

In addition, this aspect has been discussed as a limitation, particularly with regard to the potential reduced sensitivity for detecting subtle or asymptomatic recurrences.

You should acknowledge that these outcomes are exceptions and attempt to justify why this may be so.

We appreciate this important observation. The discussion has been revised to acknowledge that these outcomes should be interpreted with caution. We also explicitly recognize that non-standardized follow-up may have led to underestimation of subclinical recurrences.

Also, I would consider adding a figure of the technique as well as this will likely be the largest series of such patients to date.

As suggested, we have added some figures illustrating the main phases of the TESAR technique.
